# School-based mental health intervention for children in war-affected Burundi: a cluster randomized trial

**DOI:** 10.1186/1741-7015-12-56

**Published:** 2014-04-01

**Authors:** Wietse A Tol, Ivan H Komproe, Mark JD Jordans, Aline Ndayisaba, Prudence Ntamutumba, Heather Sipsma, Eva S Smallegange, Robert D Macy, Joop TVM de Jong

**Affiliations:** 1 Department of Mental Health, Johns Hopkins Bloomberg School of Public Health, 624 N Broadway, Hampton House R863, Baltimore, MD 21205-1996, USA; 2 Department of Research & Development, HealthNet TPO, Amsterdam, the Netherlands; 3 Faculty for Behavioral & Social Sciences, Utrecht University, Utrecht, the Netherlands; 4 Centre for Global Mental Health, Institute of Psychiatry, Kings College London, London, UK; 5 HealthNet TPO Burundi, Bujumbura, Burundi; 6 Department of Women, Children, and Family Health Science, University of Illinois at Chicago College of Nursing, Chicago, IL, USA; 7 Department of Childhood and Educational Sciences, University of Amsterdam, Amsterdam, the Netherlands; 8 International Trauma Center & Harvard School of Medicine, Boston, MA, USA; 9 Amsterdam Institute of Social Science Research, University of Amsterdam, Amsterdam, the Netherlands; 10 Boston University School of Medicine, Boston, USA; 11 Rhodes University, Grahamstown, South Africa

**Keywords:** Children, PTSD, Depression, Efficacy, Treatment, Prevention, Psychosocial intervention, Violence, War

## Abstract

**Background:**

Armed conflicts are associated with a wide range of impacts on the mental health of children and adolescents. We evaluated the effectiveness of a school-based intervention aimed at reducing symptoms of posttraumatic stress disorder, depression, and anxiety (treatment aim); and improving a sense of hope and functioning (preventive aim).

**Methods:**

We conducted a cluster randomized trial with 329 children in war-affected Burundi (aged 8 to 17 (mean 12.29 years, standard deviation 1.61); 48% girls). One group of children (n = 153) participated in a 15-session school-based intervention implemented by para-professionals, and the remaining 176 children formed a waitlist control condition. Outcomes were measured before, one week after, and three months after the intervention.

**Results:**

No main effects of the intervention were identified. However, longitudinal growth curve analyses showed six favorable and two unfavorable differences in trajectories between study conditions in interaction with several moderators. Children in the intervention condition living in larger households showed decreases on depressive symptoms and function impairment, and those living with both parents showed decreases on posttraumatic stress disorder and depressive symptoms. The groups of children in the waitlist condition showed increases in depressive symptoms. In addition, younger children and those with low levels of exposure to traumatic events in the intervention condition showed improvements on hope. Children in the waitlist condition who lived on their original or newly bought land showed improvements in hope and function impairment, whereas children in the intervention condition showed deterioration on these outcomes.

**Conclusions:**

Given inconsistent effects across studies, findings do not support this school-based intervention as a treatment for posttraumatic stress disorder and depressive symptoms in conflict-affected children. The intervention appears to have more consistent preventive benefits, but these effects are contingent upon individual (for example, age, gender) and contextual (for example, family functioning, state of conflict, displacement) variables. Results suggest the potential benefit of school-based preventive interventions particularly in post-conflict settings.

**Trial registration:**

The study was registered as ISRCTN42284825

## Background

The 2009 Machel report estimates that just over one billion children and adolescents live in countries and territories affected by armed conflict [1]. In 2011 alone, 37 armed conflicts were recorded globally, the majority in Africa (n = 15, 41%), Asia (n = 13, 35%), and the Middle East (n = 6, 16%) [2]. Epidemiological studies have shown that armed conflicts are associated with a wide range of child mental health outcomes. These may range from resilience, that is, maintained mental health in the face of adversity, to increased psychological distress and heightened prevalence of mental disorders including (symptoms of) post-traumatic stress disorder (PTSD), depression, and anxiety disorders [3].

To address the mental health burden in humanitarian settings, mental health and psychosocial support interventions are increasingly popular and consensus-based guidelines for such interventions have been developed [4,5]. These guidelines recommend implementing multi-layered packages of services, including preventive and treatment interventions, to take into account the diversity of mental health and psychosocial needs in humanitarian settings. The current study concerns a school-based mental health intervention implemented within a multi-layered package of services [6,7]. Within this package, the school-based intervention was aimed both at reducing psychological symptoms (treatment aim), as well as improving strengths and functioning in children with heightened symptomatology (preventive aim).

Despite consensus on best practices, little rigorous evidence is available on the effectiveness of child mental health interventions in humanitarian settings [8,9]. A recent meta-analysis of interventions with children affected by armed conflict in low- and middle-income countries, including six randomized controlled trials with no-intervention comparison groups, showed high heterogeneity of intervention effects across studies [10]. This high heterogeneity may be due to the diversity of interventions included in the meta-analysis (that is, specialized psychotherapeutic interventions and preventive interventions), but may also be associated with individual and contextual factors that influence intervention effects. To improve knowledge on what works for whom and under what circumstances, a crucial research direction is the identification of mediators and moderators of interventions. Mediators are variables that identify why and how interventions have effects, whereas moderators are variables that identify on whom and under what circumstances interventions have different effects [11]. Identification of mediators and moderators may assist in adapting interventions to make them more effective, or identifying the populations and contexts for which interventions are most beneficial.

This study was implemented with conflict-affected children in Burundi. Burundi is a landlocked country in the Great Lakes region of eastern Africa, with a population of 8.5 million. It is one of the poorest countries of the world, ranking 185 out of 187 countries on the Human Development Index [12]. The country has experienced cyclical ethnic violence between Hutu and Tutsi ethnic groups since 1962. The most recent violence occurred in 1993, when the killing of the first elected Hutu president sparked a civil war that killed 300,000 people and displaced 1.2 million people. Although a peace agreement was signed by most warring parties in 2000, political instability and violence continued up to the time of the study [13].

We aimed to address three research questions. First, our treatment aim: What is the effectiveness of a school-based intervention to reduce psychological symptoms (primary outcomes: PTSD, depressive, and anxiety symptoms)? Second, our preventive aim: What is the efficacy of a school-based intervention to improve hope and improve functioning (secondary outcomes)? Finally, we wanted to address the question: What are the mediators and moderators of intervention outcomes? We hypothesized that the intervention would be associated with greater reductions in symptomatology and function impairment, as well as greater improvements of a sense of hope. Our hypotheses of mediators and moderators were based on the theoretical notion of ‘ecological resilience,’ that is we expected that intervention effects would be determined by protective and risk factors at various levels of children’s social ecology (individual, family, peer, community) [14,15]. With regard to mediators, we were interested in coping and social support. We hypothesized that the intervention would be associated with larger improvements in coping and social support among children in the intervention condition, and that these improvements in turn would be associated with improvements on PTSD, depressive, and anxiety symptoms, and hope and function impairment. A systematic review on resilience and mental health in children affected by armed conflict found various studies supporting a relationship between coping and social support and lower levels of psychological symptoms, although these relations were often symptom-specific and varied by phase of conflict [16].

With regard to moderators, we hypothesized that intervention effects would vary by gender and age. Previous evaluation studies of psychosocial interventions in diverse settings have found differing effects by age and gender [17-21]. In addition, we were interested in the moderating roles of family-level variables, including household size, family connectedness, displacement status, and family composition. A longitudinal study with Afghan children found that quality of family life was an important predictor of psychological symptoms over time [22], as did a cross-sectional study in Lebanon [23]. An evaluation of a psychosocial intervention in conflict-affected areas in Indonesia found that household size influenced size of intervention effects [24]. Finally, we were interested in the potential moderating role of community-level variables, that is, social capital. In a longitudinal study with former child soldiers in Sierra Leone, a different but related variable (community acceptance) was associated with higher levels of prosocial behavior and lower levels of internalizing and externalizing symptoms over time [25]. Although the impact of armed conflict on supportive community relations has been a frequent theme in the literature on children and armed conflict [26], we are not aware of studies that have examined the role of social capital as a moderator of intervention efficacy. We hypothesized that children who perceived low levels of social capital would report stronger benefits from a psychosocial intervention, given its focus on improving supportive relations between peers.

## Methods

### Participants and screening

Research was conducted in two northwestern provinces of Burundi (Bubanza and Cibitoke), between October 2006 and June 2007. This area suffered continued violence during the period of data collection despite peace agreements in 2000 and 2003, due to the presence of remaining rebel groups. Participants were selected in three steps. First, two of the 17 provinces in Burundi were selected (Bubanza and Cibitoke), because of their continued vulnerability to political violence, the relatively homogenous socio-cultural context in these provinces, and the existence of trained human resources due to previous implementation in the area. We randomly selected either Bubanza or Cibitoke province as the intervention province. We chose to stratify at the province level to avoid risks of contamination of intervention within provinces.

**Figure 1:**
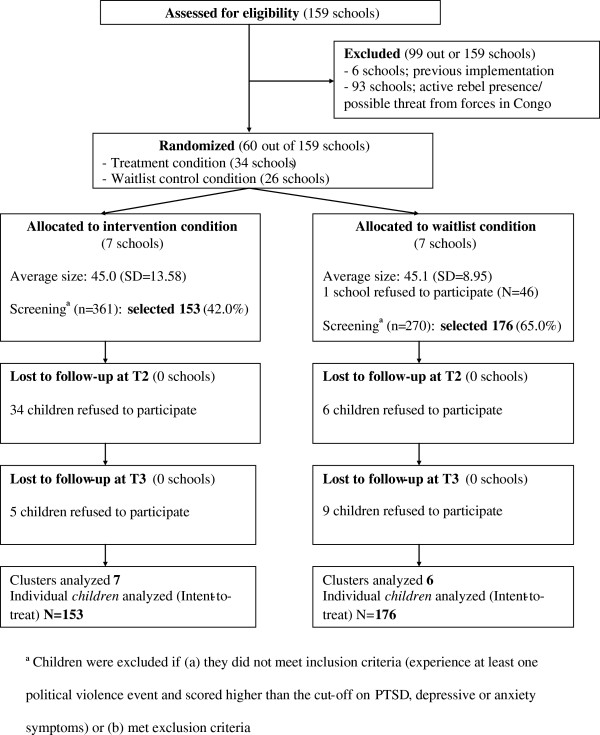
Participant flow diagram.

Second, we randomly selected schools within these provinces. We excluded all ‘communes’ (administrative unit below the province) where safety of research participants and staff could not be guaranteed, that is, forested areas populated by rebel forces (Rugazi, Musigati, Bukinyana) and communes close to restive areas in the Democratic Republic of the Congo (Mugina). We also excluded one commune in which mental health and psychosocial programs had previously been implemented. This resulted in a sampling frame of 60 schools out of the 159 schools in the two provinces (Figure 1). We chose to select 14 schools (seven per study condition) on the basis of a preceding power analysis. This power analysis was based on reported effect sizes for PTSD and depressive symptoms by Cohen *et al*. [27] and Layne *et al*. [28]. Although these studies focused on psychotherapeutic (group) interventions with trauma-affected children and our aim was to evaluate an intervention with dual aims, at the time of study design they represented the little available data on which power estimation could be based. Based on effect sizes of 1.10 for PTSD and 0.78 for depressive symptoms [27,28], a two-sided α equal to 0.02, and β equal to 0.05, we calculated that we needed a minimum of 18 and 35 children to detect changes in PTSD and depressive symptoms of similar size, respectively, per study condition. As recommended for cluster randomized trials [29], we accounted for intracluster correlation due to nested variance at the school level using the formula: n(1 + (m-1)ρ), with n = required non-corrected sample size, m = average cluster size and ρ = estimated intracluster correlation. In our sample: 35(1 + (30-1)0.1). With a power of 95%, this resulted in an appropriate sample size of 137. Allowing for attrition, we estimated that one school would represent at least 25 to 30 eligible children, hence our choice of seven schools per study condition.

Third, we screened children within schools (class 4) for eligibility using standardized checklists. Children who were exposed to at least one potentially traumatic event, and who scored above the standard cut-off on symptom checklists for either PTSD (≥11), depression (≥15), or anxiety (≥5) were included in the intervention. Criterion validity of the PTSD and depression symptom measures was examined separately against a psychiatric diagnostic interview with the Schedule for Affective Disorders and Schizophrenia for School-Age Children [30]. Results showed that the PTSD symptom checklist had an area under the curve of 0.78 with an optimum cut-off for disorder of 26 (sensitivity: 0.71; specificity: 0.83). The depression checklist had an area under the curve of 0.85, with an optimal cut-off for disorder of 19 (sensitivity: 0.64; specificity: 0.88). However, because our interest was to evaluate the effects of the intervention on both preventive and treatment aims, the original cut-off scores were retained.

### Intervention

The classroom-based intervention (CBI) was part of a multi-layered care package implemented in schools that also included universal preventive activities (for example, structured social activities with those not screened into the CBI) and provision of mental health treatments (for example, psychosocial counseling and referral to mental health specialists) [7,31]. Within this care package, CBI was aimed at decreasing psychological symptoms and strengthening protective factors in children at risk, that is, those displaying heightened psychological symptoms. CBI entailed 15 sessions over five weeks implemented by locally identified non-specialized facilitators trained and supervised in implementing the intervention for one year prior to the study. Facilitators had at least a high school diploma and were selected for their affinity and capacity to work with children as demonstrated in role-plays and interviews. The manualized intervention consisted of cognitive behavioral techniques (psychoeducation, strengthening coping, and discussion of past traumatic events through drawing) and creative expressive elements (cooperative games, structured movement, music, drama, and dance) with groups of around 15 children. The intervention was structured to have specific themes across sessions with the following foci: information, safety, and control in week 1 (sessions 1 to 3); stabilization, awareness, and self-esteem in week 2 (sessions 4 to 6); the trauma narrative in week 3 (sessions 7 to 9); resource identification and coping skills in week 4 (sessions 10 to 12); and reconnection with the social context and future planning in week 5 (sessions 13 to 15). Each individual session was structured into four parts, starting and ending with structured movement, songs, and dance with the use of a ‘parachute’ (a large circular colored piece of fabric). The second part was based on a ‘central activity’ focused on the main theme of that week (for example, a drama exercise to identify social supports in the environment, or drawing of traumatic events), and the third part was a ‘cooperative game’ (that is, a game in which all children had to participate to promote group cohesion) [32].

### Instruments

Instruments were selected based on previously conducted qualitative research in northwestern Burundi (data not shown, see [33]). This qualitative research entailed 14 focus group discussions, 40 semi-structured interviews with children and caregivers identified as affected by the civil war, and 32 key informant interviews (including traditional or religious healers, teachers, community health workers, clergy, and staff of organizations assisting war-affected children). Content analysis of verbatim recorded data evidenced an interrelated set of children’s problems, including war-related problems at the individual, family (large-scale loss of parents, abuse by foster families, increased family conflicts over land), peer (distrust between Hutu and Tutsi peers), and community levels (loss of social solidarity, increased accusations of supernatural harm, ethnic hate and distrust) [15]. Often mentioned psychological problems included fears, sadness or despair, being reminded of bad events, loneliness, inactivity, anger and aggression, and grief-related problems. Based on this information, we selected standardized measures for PTSD, depressive, and anxiety symptoms (primary outcomes), and added context-specific items to the interviews.

Standardized measures were translated using a five-step method for preparation of instruments in transcultural research, which included bilingual translation, independent bi-lingual conceptual review, blind-back translation, focus groups, and piloting with target population [34]. Test-retest reliability (TRR) (Spearman-Brown correlation) was assessed over a two-week period with a convenience sample of 15 children.

We used the *Child Posttraumatic Symptom Scale* (17 items; four-point scale; range 0 to 51; internal reliability (IR, Cronbach Alpha) = 0.84; TRR = 0.59, *P* = 0.032) to assess PTSD symptoms [35]. Depressive symptoms were assessed using the *Depression Self-Rating Scale* (18 items; three-point scale; range 0 to 36; IR = 0.72; TRR = 0.88, *P* <0.000) [36]. We measured anxiety using the five-item version of the *Screen for Anxiety Related Emotional Disorders*[37]. Although pre-trial piloting showed acceptable IR, Cronbach Alpha in the study was low (0.28), so we did not consider these items in further analyses.

To assess sense of hope, we used the *Children’s Hope Scale*[38] (six items; six-point scale; range 6 to 36; IR = 0.70; TRR = 0.95, *P* <0.000). In this measure, hope is operationalized as a similar but different construct to self-esteem, and consists of agency (that is, the perception that children can initiate and sustain action towards a certain goal) and pathways (that is, perceived capability to produce routes to those goals). We also constructed a measure to assess function impairment using a previously applied mixed-methods approach that involved brief participant observation, collection of diaries, and focus groups [39]. The function impairment measure consisted of nine items and asked about impairment in such daily activities as hygiene, playing, household chores, studying, and religious activities (four-point scale; range 9 to 36; IR = 0.80; TRR = 0.73, *P* = 0.001).

#### Mediators

We measured coping using the child-rated *Kidcope*[40]. The *Kidcope* contains 15 questions concerning use of 10 different coping strategies and satisfaction with used coping strategies. From this scale we derived a coping repertoire index, by summing the amount of coping strategies that were endorsed by children (dichotomous; range 0 to 15; TRR = 0.75, *P* = 0.003); and a coping satisfaction (three-point scale; range 15 to 45; IR = 0.68; TRR = 0.82, *P* <0.000). Social support was assessed with the *Social Support Inventory Scheme*[41]. This measure asks children to list up to five people from whom they receive support and asks, for each of these people, whether they provided material (for example, giving food, clothes, helping with school fee), emotional (for example, cheering up, listening or attending to problems), guidance (for example, providing advice, teaching something), or play social support (singing, dancing, storytelling to feel better). For each support type, the child answers yes or no. From this measure we calculated a total social support measure by adding up the different types of support received (range 0 to 20; TRR not assessed).

#### Moderators

Gender, age, displacement status, household size, and family composition were all assessed through one-item questions as part of the demographics questionnaire. To measure exposure to traumatic events (11 items, dichotomous, range 0 to 11), we constructed a checklist locally through free listing. This consisted of asking 23 staff of implementing organization HealthNet TPO Burundi to list adverse events children may be exposed to as part of the armed conflict. We selected the traumatic events that were mentioned by five people or more for inclusion in the checklist. Finally, social capital was assessed with a locally constructed measure modeled after the *Short Adapted Social Capital Assessment Tool*[42]. Based on our qualitative data in which participants described how community ties were damaged by the conflict, we selected two items from the cognitive dimension of this instrument (trust in other children, children getting along), and added items asking about social solidarity, sense of community, sharing of child care, and perception of members’ behavior towards each other (seven items; four-point scale; range 7 to 28; IR = 0.72).

### Procedures

All measures were interviewer-administered in private settings within schools, by interviewers trained over a four-week period. Instruments were applied one week before the intervention (T1), one week after the intervention (T2), and three months after the intervention (T3). After a complete description of the study to the participants, written informed consent was obtained from both children and parents. Ethical permission was granted by the Internal Review Board of the VU University Amsterdam. In addition, we obtained local permission from governors of provinces, all school principals, and village leaders in the areas where schools were located. One school in the waitlist condition discontinued participation after the baseline assessment (Figure 1).

### Data analysis

We assessed the comparability of study conditions (demographic characteristics, scores on moderators and mediators at baseline) by applying χ^2^ with continuity correction or Fisher exact test for frequencies, and independent-sample t-tests for continuous measures.

Analyses of crude changes on the outcome measures, that is, mean changes not corrected for clustered variance at the school level, were conducted by computing pure change scores between baseline and follow-up scores (T1 to T3 for boys and girls separately on an intent-to-treat basis (last value carried forward). These pure change scores were compared using independent-sample t-tests.

To correct for clustering and examine the role of moderators and mediators, longitudinal changes on outcome measures were examined through latent growth curve modeling in a structural equation modeling framework [43]. Conditional growth models were used to estimate the intervention main effect and to model moderating effects while controlling for main effects accordingly. All models controlled for clustering at the school level. Latent growth curve modeling was conducted in two steps. First, we modeled growth curves, using 0, 6 and 20 weeks as time points, and estimated the effect of intervention on changes over time. Second, we added moderators and their main effects to explore potential variations in intervention effects. All growth models used maximum likelihood estimation to model all data available for dependent variables. Latent growth curve modeling was conducted using MPlus 4.21 [44].

## Results

### Characteristics at baseline

We compared study conditions at baseline on demographic characteristics and scores on the outcomes measures, mediators and moderators (Tables 1 and 2). Children in the waitlist condition were exposed to fewer categories of traumatic events (0.5 type of event), and reported lower cognitive social capital at baseline. Children in the treatment condition had lower depressive scores at baseline and reported fewer coping strategies, smaller satisfaction with coping, and less social support than their counterparts in the waitlist condition.

**Table 1:** Clusters and baseline comparison of scores on moderators

			**Intervention condition N (%)**^**a**^	**Waitlist condition N (%)**^**a**^	**Chi-square (df); *P***
Clusters	Number		7	6	
	Average Size		21.9	29.3	
	Total N (N = 329)		153	176	
Moderators	Gender	Boys	77 (50.3)	94 (53.4)	0.457 (1); 0.509
		Girls	76 (49.7)	82 (46.6)	
	Age (years)	<11	32 (20.9)	55 (31.3)	5.507 (2); 0.064
		12 to 15	117 (76.5)	119 (67.6)	
		>16	3 (2.0)	1 (0.6)	
		Missing	1 (0.7)	1 (0.6)	
	Household size		Mean = 6.9; SD = 2.30	Mean = 6.8; SD = 2.17	t = 0.435 (325); 0.664
	Displacement status	Original village	76 (49.7)	77 (43.8)	5.116 (4); 0.276
		Other village	37 (24.2)	51 (29.0)	
		Refugee camp	29 (19.0)	26 (14.8)	
		Bought new land	8 (5.2)	18 (10.2)	
		Other	3 (2.0)	2 (1.1)	
		Missing	0 (0.0)	2 (1.1)	
	Family composition	Two parents in household	103 (67.3)	118 (67.4)	1.058 (3); 0.787
		One parent in household	31 (20.3)	36 (20.6)	
		Other type of adult caregiver	15 (9.8)	19 (10.9)	
		No adult caregiver	4 (2.6)	2 (1.1)	
	Traumatic events		Mean = 4.6, SD = 2.05	Mean = 4.1, SD = 2.05	t = 1.996 (327); 0.047*
	Social capital		Mean = 18.4, SD = 3.63	Mean = 17.0, SD = 2.92	t = 3.924 (327); 0.000**

^a^Percentages may not add up to 100 due to rounding off. df, degrees of freedom; SD, standard deviation; *: statistically significant at α 0.01-0.05, **: statistically significant at α <0.01.

**Table 2:** Baseline comparisons of scores on outcome measures and mediators

		**Intervention condition**		**Waitlist condition**		**T (df)**	***P***	**ICC**
		**(N = 153)**		**(N = 176)**				
		**Mean**	**SD**	**Mean**	**SD**			
Outcomes	PTSD symptoms	15.62	9.42	16.30	7.35	−0.738 (327)	0.461	0.035
	Depressive symptoms	9.97	4.82	11.28	5.08	−2.396 (327)	0.017*	0.036
	Hope	15.97	5.77	15.03	5.81	1.471 (327)	0.142	0.038
	Function impairment	13.97	5.01	14.49	5.12	−0.934 (327)	0.351	0.035
Mediators	Coping repertoire	7.72	2.57	8.92	2.40	−4.354 (325)	0.000**	0.036
	Coping satisfaction	17.68	7.01	19.66	5.84	−2.793 (325)	0.006**	0.036
	Social support	10.51	5.10	12.63	4.46	−4.024 (327)	0.000**	0.031

df, degrees of freedom; ICC, intra cluster correlation; PTSD, posttraumatic stress disorder; SD, standard deviation; *: statistically significant at α 0.01-0.05, **: statistically significant at α <0.01.

We compared baseline mean values for children for whom complete follow-up was achieved (that is, T1, T2, and T3 participation) with children who dropped out at T2 (n = 40, 12.2%) or T3 (n = 14, 4.3%). No statistically significant differences were found with regard to exposure to traumatic events, depressive symptoms, hope, function impairment, or coping satisfaction. However, completers had lower baseline levels of PTSD (13.7 versus 16.4 in completers, T = 2.27, *P* = 0.024), higher levels of social capital (18.5 versus 17.5, T = -2.05, *P* = 0.042), and lower levels of total social support (9.9 versus 12.0, T = 2.64, *P* = 0.010).

### Comparison of changes over time

Per illustration, Table 3 provides crude (unadjusted) t-test comparisons between the treatment and waitlist condition of changes on mental health outcomes and putative mediators over time by gender. No statistically significant differences were found for mean changes between study conditions on outcomes and mediators.

**Table 3:** Unadjusted comparison of differences in mean changes (baseline to three-month follow-up)

		**Boys**						**Girls**					
		**Intervention (N = 77)**		**Waitlist (N = 94)**				**Intervention (N = 76)**		**Waitlist (N = 82)**			
		**ΔT1 to T3**	**SD**	**ΔT1 to T3**	**SD**	**T; df = 169**	***P***	**ΔT1 to T3**	**SD**	**ΔT1 to T3**	**SD**	**T; df = 156**	***P***
		**Mean**		**Mean**				**Mean**		**Mean**			
Outcomes	PTSD symptoms	5.68	9.65	4.85	8.56	−0.597	0.551	5.85	10.97	5.18	11.46	−0.380	0.704
	Depressive symptoms	1.42	4.61	2.58	5.60	1.451	0.149	2.21	5.42	3.00	6.00	0.864	0.389
	Hope	−1.03	6.71	−0.47	6.59	0.546	0.586	−2.97	6.27	−1.93	6.17	1.058	0.292
	Function impairment	1.13	4.75	0.39	6.18	-0.865	0.388	1.76	5.06	1.95	4.93	0.237	0.813
Mediators	Coping repertoire	0.23	3.38	0.69	3.17	0.915	0.362	0.22	3.57	−0.05	3.81	−0.454	0.651
	Coping satisfaction	1.18	7.85	0.55	8.03	−0.514	0.608	1.48	7.41	0.89	8.38	−0.465	0.642
	Social support	−0.01	4.12	−1.32	4.85	−1.866	0.056	0.24	5.58	−0.62	5.67	−0.959	0.339

df, degrees of freedom; PTSD, posttraumatic stress disorder; SD, standard deviation.

#### Main effects

For a true test of our hypothesis, we conducted latent growth curve modeling of changes over time that corrected for nested variance. As can be seen in Table 4, these analyses confirmed the lack of main effects of the intervention on outcome measures. Given that we did not find significant changes on the mediators over time, a first condition for establishing potential mediation effects, we did not pursue further analysis of mediation effects.

**Table 4:** Model estimates of longitudinal changes on outcome measures

**Outcome**	**Intercept**^**a**^		**Slope: Main effect**^**b**^		**Slope: Interaction with gender**^**c**^		**Slope: Interaction with age**^**d**^		**Slope: Interaction with household size**		**Slope: Interaction with exposure**		**Slope: Interaction with social capital**		**Slope: Interaction with family composition**		**Slope: Interaction with displacement status**	
	**Est**	**SE**	**Est**	**SE**	**Est**	**SE**	**Est**	**SE**	**Est**	**SE**	**Est**	**SE**	**Est**	**SE**	**Est**	**SE**	**Est**	**SE**
PTSD symptoms	−0.378	1.776	−0.073	0.147	0.013	0.109	0.053	0.040	−0.035	0.022	−0.015	0.017	−0.027	0.018	−0.197	0.064**	0.069	0.068
Depressive symptoms	−0.651	0.782	−0.008	0.062	−0.001	0.055	0.029	0.022	−0.018	0.007*	0.009	0.012	0.007	0.008	−0.110	0.042**	0.024	0.038
Hope	1.063	0.660	0.065	0.073	−0.066	0.058	−0.056	0.025*	0.004	0.009	−0.028	0.011*	0.018	0.012	0.024	0.056	−0.196	0.064**
Function impairment	−0.256	1.060	−0.035	0.045	−0.036	0.053	0.027	0.015	−0.011	0.006*	−0.009	0.007	0.008	0.005	0.034	0.037	0.116	0.030**

^a^Average score at baseline corrected for clustering; ^b^change on outcome measure over 0, 6 and 20 weeks; ^c^a negative estimate indicates decreases in the outcome for boys; ^d^a negative estimate indicates decreases in the outcome for every year older; *: statistically significant at α 0.01−0.05, **: statistically significant at α <0.01. Est, estimate; PTSD, posttraumatic stress disorder; SE, standard error.

#### Moderators

Next, we compared trajectories of outcome measures between study conditions, while taking into account interaction effects with potential moderators of intervention. These analyses showed several instances where changes on outcome measures over time were different between study conditions in interaction with moderators. For ease of reading, these effects are summarized in Table 5. First, the effect of household size on depressive symptom and function impairment trajectories was statistically significantly different between study conditions. Children in the intervention condition living in larger households showed greater improvements for both depressive symptom and function impairment trajectories, whereas there were no effects of household size on either trajectory in the waitlist control condition (depressive symptoms: intervention condition estimate = -0.016, SE = 0.004, Z = -3.640 (z-test and waitlist control condition estimate = 0.002, SE = 0.004, Z = 0.509; function impairment: intervention condition estimate = -0.009, SE = 0.005, Z = -2.050 and waitlist control condition estimate = 0.002, SE = 0.005, Z = 0.369). In addition, we found different trajectories between study conditions for PTSD and depressive symptoms when analyzing the interaction with family composition. In the intervention condition, living with both parents was associated with statistically significant decreases in PTSD and depressive symptoms (PTSD estimate = -0.158, SE = 0.036, Z = -4.348; depressive symptoms estimate = -0.069, SE = 0.020, Z = -3.371). There was no association between living with both parents and PTSD in the waitlist condition (estimate = 0.039, SE = 0.036, Z = 1.080); however, living with both parents was associated with significant increases in depressive symptoms over time in the waitlist control condition (estimate = 0.041, SE = 0.020, Z = 2.001).

**Table 5:** Overview of findings

**Outcome**	**More favorable longitudinal trajectory**	**Moderator**
PTSD	Intervention condition	Family composition (living with both parents)
Depression	Intervention condition	Family composition (living with both parents)
	Intervention condition	More members in household
Hope	Intervention condition	Younger age
	Intervention condition	Lower trauma exposure
	Control condition	Displacement^a^
Functioning	Intervention condition	More members in household
	Control condition	Displacement

^a^More favorable outcomes in waitlisted children who lived on their own land or newly bought land. PTSD, posttraumatic stress disorder.

Second, for the outcome, hope, we found statistically significant different trajectories between study conditions in interaction with three moderators: age, exposure, and displacement status. With regard to age, in the intervention condition younger age was associated with increased hope, whereas there was no association between age and hope in the waitlist condition (intervention condition estimate = -0.060, SE = 0.009, Z = -6.639; waitlist control condition estimate = -0.004, SE = 0.013, Z = -0.355). Hope also increased among children with fewer exposures in the intervention condition (estimate = -0.029, SE = 0.009, Z = -3.237), but there was no association between exposures and hope in the waitlist control condition (estimate = -0.001, SE = 0.009, Z = -0.142). Hope, however, also increased among children living in their original villages or who had bought new land in the waitlist control condition, but decreased over time among these children in the intervention condition (intervention condition estimate = -0.116, SE = 0.025, Z = -4.607; waitlist control condition estimate = 0.080, SE = 0.025, Z = 3.150).

Third, we also found differences in trajectories for function impairment by displacement status. In the intervention condition, function impairment increased for children living in their original villages or who had bought new land (estimate = 0.060, SE = 0.018, Z = 3.250) but decreased in the waitlist control condition (estimate = -0.056, SE = 0.018, Z = -3.062).

## Discussion

This study was aimed at identifying intervention outcomes, and mediators and moderators of a school-based mental health intervention for children in war-affected Burundi. The intervention was aimed both at the reduction of PTSD, depressive, and anxiety symptoms (treatment aim, primary outcomes), as well as the improvement of hope and functioning (preventive aim, secondary outcomes). We did not find any main effects on the primary and secondary outcome measures, that is, for either the treatment or preventive aims. Therefore, mediation analyses were not performed. However, we did find eight differences in longitudinal trajectories between study conditions in interaction with a number of moderators. These moderation effects are challenging to interpret clinically, as commonly applied effect size calculations do not take clustered variance into account and may obscure subgroup findings. However, moderation effects generally applied to larger groups of children in our sample (for example, children living with both parents, 67.2% of the sample; children with larger households, 54.3% had seven members or more; children with lower trauma exposure, 21.6% reported two or fewer events; children living in their original village or bought land, 54.8%).

Before discussing these findings in more detail, we highlight limitations of the study. First, we found statistically significant differences between the study conditions at baseline, which may have been the result of randomization by province before randomization of schools. It is unknown how these differences may have impacted findings on intervention effects, given that children in the intervention condition were doing better in some aspects (that is, lower depressive symptoms, higher social capital) but worse in other aspects (that is, higher trauma exposure, fewer coping strategies and coping satisfaction, lower social support) at baseline. Differences may also have been associated with the fact that relatively few schools were randomized, which the analyses controlled for. In addition, differences were found mainly for variables for which we did not find significant intervention effects, with the exception of trauma exposure and depressive symptoms (three of eight differences identified between study conditions). Second, drop-out may have affected study findings. As with the baseline differences between study conditions, it is difficult to say how these findings may have impacted study findings, because study completers had lower levels of PTSD and higher levels of social capital, but lower levels of social support. Third, we had to exclude our measure of anxiety due to low IR of the measure. Although our other measures had good IR and TRR, unmeasured factors that contributed to low IR of the anxiety measure may have impacted our other instruments in ways we did not measure. Fourth, our research assessors were working independently from the implementation team, but were not blinded to study condition because they had to visit schools to interview children. We emphasized in training of assessors that an objective evaluation was crucial. However, the lack of blinding assessors may have biased findings in favor of the intervention. Strengths of the study include the detailed translation and mixed methods procedures to prepare instruments, the inclusion of a broader range of measures to assess different aspects of mental health (symptoms, hope, functioning), the inclusion of a follow-up assessment three months after the intervention, and the examination of moderators.

Our current findings from Burundi add to a number of recent studies that have rigorously evaluated this school-based intervention with conflict-affected populations in Indonesia, Nepal, the occupied Palestinian territories, and Sri Lanka. Collectively, these studies provide emerging evidence-based answers to important questions on the practical benefit of this and similar interventions. First, given inconsistent results on primary outcome measures across settings, it now seems that this school-based intervention should not be recommended as a treatment for PTSD, depressive, and anxiety symptoms. Although the school-based intervention was associated with reductions in these symptoms in some settings (such as girls in Indonesia, boys and children with less ongoing trauma exposure in Sri Lanka, and children with both parents in Burundi), it was not associated with improvements in these symptoms in Nepal and the occupied Palestinian territories, and was associated with unfavorable effects on PTSD symptoms in Sri Lankan girls and older children in the occupied Palestinian territories. Rather, recent World Health Organization guidelines for non-specialized health settings in low- and middle-income countries recommend cognitive behavioral treatments with a trauma focus and eye movement desensitization reprocessing as treatments for PTSD in children and adolescents [45]. Although this school-based intervention incorporated cognitive behavioral elements (for example, working on coping skills, psycho-education, some discussion of trauma-related material through drawings), it did not comprise consistent trauma exposure, memory, or cognitive processing when compared to cognitive behavioral treatments with a trauma focus or to eye movement desensitization reprocessing. On the basis of findings in Indonesia and Sri Lanka, we previously argued that this intervention may nevertheless have a place in a spectrum of treatment options: despite smaller main effects, it can reach more children with fewer resources. Given the inconsistent results across settings, however, it may be better to start with World Health Organization recommendations for treatment of PTSD in children and adolescents. A remaining key research question here is how existing evidence-based interventions, often tested in more highly resourced research settings, can be effectively disseminated and implemented in real-world health-care settings [46-48].

When considering the benefit of the school-based intervention as a preventive intervention, that is, in strengthening resilience processes in conflict-affected children, the intervention seems to have more consistent results across settings. Intervention effects were found for hope, positive coping, social support, and function impairment in Indonesia; hope and prosocial behavior in Nepal; hope in Sri Lanka; hope and a range of other strengths in the occupied Palestinian territories; and hope and function impairment in this study. However, in this study in Burundi, displaced children in the intervention condition had worse trajectories on hope and function impairment. Further adaptation of this school-based intervention may focus on removing the trauma-focused elements (see [49]) and implementing it only as a preventive tool, as well as concentrating on more active involvement of families and communities. An important future direction would then be to examine whether changes in strengths in the shorter term translate to improvements in psychological symptoms and overall wellbeing and development in the longer term.

An important question concerns the differential intervention effects (both treatment and preventive) by gender, age, and a variety of contextual factors. We feel results across studies may best be explained from the theoretical perspective of ecological resilience. This theoretical framework aims to explain children’s mental health by examining which resources (strengths) are available in children and their social contexts, at family, peer, and community levels. From this perspective, the complex differential effects of the intervention may be clarified by the extent to which the resources in children’s environment may interact with intervention activities. The most positive intervention effects were observed in Central Sulawesi, Indonesia, which may be explained by the fact that children there were still living in generally supportive families and communities - although tension remained between the communities after the conflict [15,33]. In Indonesia, the children that benefited most from the intervention were the children who were socially more isolated [24]. It appears that in settings that are more volatile (for example, Burundi, Sri Lanka), the resilience of children that live in particularly stressful conditions may actually be undermined by the intervention (for example, girls and children exposed to higher levels of ongoing stressors in Sri Lanka, male adolescents in the occupied Palestinian territories, displaced children in Burundi). In these settings, a preliminary intervention recommendation would be to have a clearer separation between intervention aims and to implement interventions in more homogenous groups, so that only the positive preventive effects may be achieved with children in more stable situations, and more intensive treatments may reach those who are more vulnerable. Detailed pre-intervention assessments seem crucial to identify who the particularly socially vulnerable children may be.

From a scientific point of view, the identified differential effects from our studies call for a more detailed look at how context interacts with intervention effects in conflict-affected settings, particularly for evaluations of preventive interventions. This would require an adaptation of the randomized controlled trial (for example, with a cohort multiple randomized controlled trial design) to encompass the multi-disciplinary examination of family-, peer-, and community-level variables before and during implementation of interventions, and the study of how such variables interact over the longer term with psychological symptoms. Following advances that have been made in the field of treatment of PTSD symptoms, such scientific developments would aid in strengthening efforts to prevent long-lasting psychological symptoms and promote mental health in children affected by armed conflict.

Furthermore, a promising research direction for preventive efforts could be to intervene in earlier developmental periods, when parenting skills and parental mental health can be enhanced before negative patterns set in [50]. Given that development proceeds sequentially (that is, skills learned later in life build on skills learned earlier), researchers focusing on children growing up in adversity have emphasized the early childhood period as a particularly cost-effective period for intervention [51,52]. For example, in a randomized controlled trial with 87 displaced mother-child dyads (mean age of children, 5.5 years), Dybdahl found that a group intervention aimed at strengthening parental involvement, support, and education in mothers had promising benefits for their children [53].

## Conclusions

Our evaluation of a school-based mental health intervention with children affected by armed conflict showed both benefits and unfavorable effects in interaction with moderators that included age, exposure to potentially traumatic events, family composition, size of household, and displacement status. For treatment purposes, other interventions may be more suitable. For preventive interventions, further multi-disciplinary studies are required to study how intervention at the family, peer, and community levels may best promote mental health and development, and prevent occurrence of psychological symptoms over time.

## Abbreviations

CBI: classroom-based intervention; IR: internal reliability; PTSD: post-traumatic stress disorder; TRR: test-retest reliability.

## Competing interests

The authors declare that they have no competing interests.

## Author contributions

WT, IK, MJ, RM, JdJ designed the study. WT, AN, ES supervised data collection. MJ and PN supervised implementation of the intervention. WT, HS analyzed the data, with critical inputs from IK, MJ, JdJ. WT wrote a first draft of the manuscript. WT, IK, MJ, AN, PN, HS, ES, RM, JdJ revised the manuscript for important intellectual content. All authors read and approved the final manuscript.

## Pre-publication history

The pre-publication history for this paper can be accessed here:

http://www.biomedcentral.com/1741-7015/12/56/prepub
